# Draft genome sequence of Taiwanese pear (*Pyrus pyrifolia*)

**DOI:** 10.1016/j.dib.2018.06.056

**Published:** 2018-06-26

**Authors:** Yoshihiro Takemura, Fumio Tamura

**Affiliations:** Faculty of Agriculture, Tottori University, Koyama, Tottori 680-8553, Japan

## Abstract

Pear (genus *Pyrus*) is one of the oldest temperate fruit crops, of which at least 22 primary species are recognized. This article documents the public availability of the partial draft genome sequence data of the Taiwanese pear ‘Hengshanli’ that is less dormant during the winter season. This dataset may be used to prepare molecular markers for the breeding of new cultivars that are subjected to chilling at low temperatures to overcome endodormancy. This data will also help analyze the process of evolution in the *Pyrus* species. We sequenced paired-end libraries using Illumina HiSeq. 2500 and generated approximately 210M reads. Data on the draft genome obtained in this study has been deposited to the DNA Data Bank of Japan (DDBJ). The read data were submitted to the DDBJ Read Archive (BioProject: PRJDB6877, BioSample: SAMD00117052).

**Specifications Table**

**Table:** 

Subject area	Biology
More specific subject area	Horticultural science, Genomics
Type of data	Genome sequence data
How data was acquired	Illumina HiSeq. 2500 Next Generation Sequencing Platforms
Data format	Raw sequencing reads
Experimental factors	Draft genome sequence of Taiwanese pear was performed by using Illumina HiSeq.
Experimental features	Sequencing was performed according to Illumina sequencing protocols for DNA-seq
Data source location	Shizuoka, Japan
Data accessibility	The draft genome sequences of Taiwanese pear (*Pyrus pyrifolia*) have been deposited in the DNA Data Bank of Japan (DDBJ) under the DDBJ Read Archive (BioProject: PRJDB6877 (https://ddbj.nig.ac.jp/BPSearch/bioproject?acc=PRJDB6877), BioSample: SAMD00117052).

**Value of the data**

• This dataset may be used to compare between the genomic data of other *Pyrus* species.
• It will also be useful to prepare molecular markers for the breeding of new cultivars that are subjected to chilling at low temperatures for breaking endodormancy, because Taiwanese pear Hengshanli is less dormant during the winter season.
• This data will further be valuable for more detailed study of evolutionary processes occurring in *Pyrus* plants.

## 1 Data

Pear (*Pyrus* spp.) belongs to the family Rosaceae, and is generally divided into two groups: Occidental pears (European pears) and oriental pears (Asiatic pears) [1], [2]. Asiatic pears are grown extensively in Mainland China, Japan, Korea, and Taiwan [3]. We present a partial draft genome sequence of the Taiwanese pear Hengshanli, a cultivar that is extensively grown in Taiwan. We sequenced paired-end libraries using Illumina HiSeq. 2500 and generated approximately 210M reads. This data will be useful to prepare molecular markers for breeding new cultivars that are subjected to chilling at low temperatures for breaking endodormancy, because Taiwanese pear Hengshanli is less dormant during the winter season [4], [5]. Additionally, this data will also be valuable for a more detailed study of the evolutionary processes occurring in *Pyrus* species by providing genomic data for a comparative analysis with Chinese pear [6] and European pear [7] that have already been analyzed.

## 2 Experimental design, materials, and methods

### 2.1 DNA extraction

Genomic DNA was extracted from the young leaves of the Taiwanese pear Hengshanli, using a modified version of the cetyltrimethylammonium bromide (CTAB) protocol [8]. Quality and quantity of DNA were checked by Agilent 2200 TapeStation (Agilent Technologies, Santa Clara, USA).

### 2.2 Sequencing

The extracted genomic DNA was subjected to preparation of a paired-end library for genome sequencing using the Illumina HiSeq. 2500. Genomic DNA libraries were constructed using the TruSeq Nano DNA Library Prep Kit (Illumina, Cat FC-121-4001) according to the manufacturer׳s protocol.

### 2.3 Data accessibility

The read data were submitted to the DDBJ Read Archive (BioProject: PSUB008444, BioSample: SSUB009289).
