# Supplementary material for: Draft genome sequence of Taiwanese pear (Pyrus pyrifolia)
Source: Data Brief. 2018 Jun 26;19:1871–3. doi: 10.1016/j.dib.2018.06.056 (PMC6141270; doi:10.1016/j.dib.2018.06.056)
Supplement: Supplementary file 1 — Supplementary material [file mmc1.docx]

**Conflict of Interest Form**

The authors have no conflicts of interest directly relevant to the content of this article.
